# Chlorambucil-induced cytomegalovirus infection: a case report

**DOI:** 10.1186/1752-1947-8-280

**Published:** 2014-08-20

**Authors:** María Thiscal López-Lluva, María Dolores Sanchez de la Nieta-García, Jesús Piqueras-Flores, Minerva Arambarri-Segura, Alberto Martínez-Calero, Francisco Rivera-Hernández

**Affiliations:** 1 Servicio de Cardiología. Hospital General Universitario de Ciudad Real, Calle del Obispo Rafael Torija s/n, 13005 Ciudad Real, España; 2 Servicio de Nefrología. Hospital General Universitario de Ciudad Real, Calle del Obispo Rafael Torija s/n, 13005 Ciudad Real, España

## Abstract

**Introduction:**

Chlorambucil is an alkylating agent used in combination with prednisolone for the treatment of idiopathic membranous nephropathy. Although chlorambucil is generally well-tolerated, it is a myelosuppresive drug that can cause several infections.

**Case presentation:**

We report the case of an 81-year-old Caucasian male presenting with idiopathic membranous nephropathy who developed fever, cough, dyspnea, pulmonary infiltrates, and abdominal pain shortly after the initiation of treatment with chlorambucil and corticosteroids for nephropathy. Virology tests for infectious diseases revealed a recent cytomegalovirus infection. Antiviral treatment (ganciclovir) resulted in full remission.

**Conclusions:**

Cytomegalovirus infection should be considered in the differential diagnosis of respiratory symptoms and pulmonary infiltrates in patients treated with chlorambucil for nephrotic syndrome.

## Introduction

Chlorambucil (CBL) is an alkylating agent used in combination with prednisolone for the treatment of idiopathic membranous nephropathy. Although CLB is generally well-tolerated, it is a myelosuppresive drug that can cause several infections. We report such a case of idiopathic membranous nephropathy that developed a pulmonary cytomegalovirus (CMV) infection shortly after treatment with chlorambucil.

## Case presentation

An 81-year-old Caucasian male presented to our hospital with a 10-day history of fever and non-productive cough. He also complained of abdominal pain, malaise, and weight loss.

His past medical history included hypertensive cardiomyopathy. He was diagnosed with nephrotic syndrome several months previously when a renal biopsy showed changes of membranous nephropathy stage III. Three months after this diagnosis, he was admitted to our hospital due to progressive worsening of renal function and persistent nephrotic syndrome. A laboratory test, chest radiograph, and an abdominal ultrasound scan ruled out secondary causes of membranous nephropathy and treatment with CBL 10mg and methylprednisolone 0.5mg/kg daily was started. Twenty-two days after the initiation of this treatment, he presented to our hospital with fever and non-productive cough and was admitted.

On initial assessment his temperature was 38°C, his blood pressure was 125/75mmHg, and his cardiac frequency was 105 beats per minute. His oxygen saturation level was 93%. There were no oral lesions. An examination of the lungs revealed widespread rhonchi. A cardiac examination demonstrated a regular rhythm and no murmurs. His abdomen was soft, not distended, but painful at the epigastrium.

**Figure 1:**
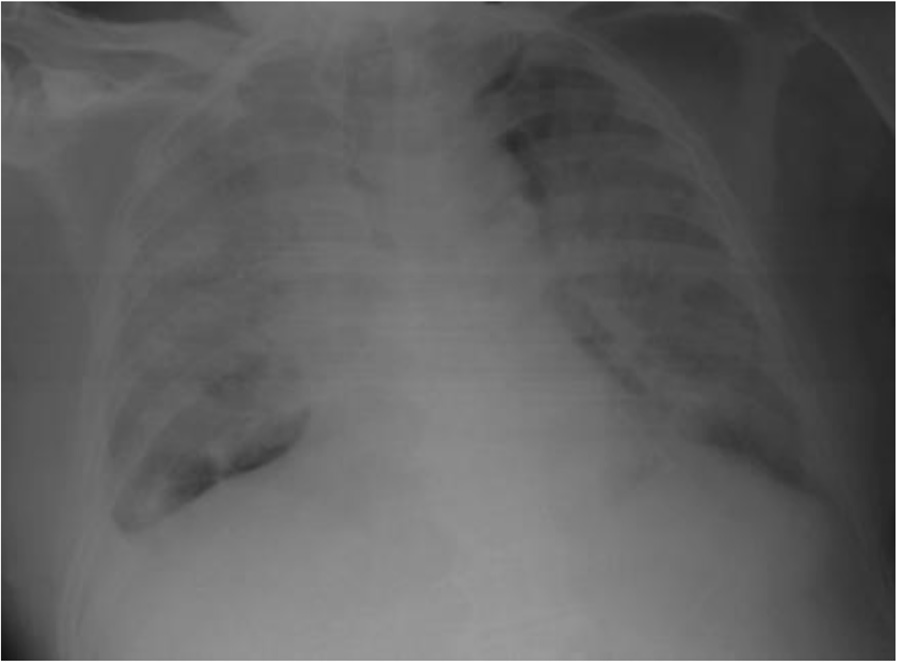
Bilateral diffuse interstitial lung disease with small bilateral pleural effusion.

**Figure 2:**
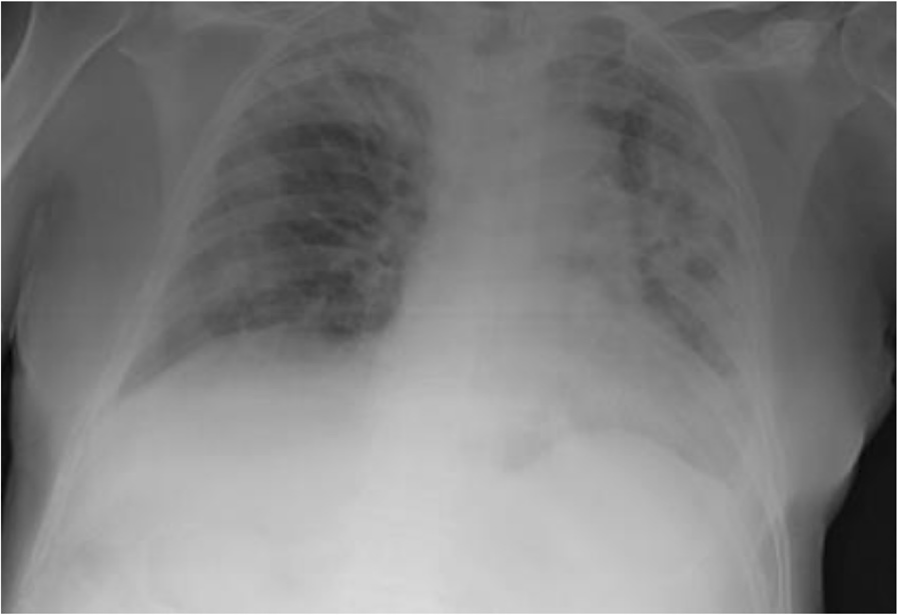
Irregular, multifocal, patchy consolidations.

His laboratory tests showed a white blood cell count of 2.3×109/L, neutrophils at 82%, a hemoglobin level of 11.8g/dL, platelets at 96×109/L, lactate dehydrogenase at 965U/L, a serum creatinine of 2.8mg/dL, urea at 164mg/dL, total proteins at 4.1g/dL, albumin at 2.6g/dL aspartate transaminase at 74U/L, alanine transaminase at 98U/L, gamma-glutamyltransferase at 255U/L, cholesterol at 219mg/dL, and triglyceride at 416mg/dL. His procalcitonin level was lower than 0.5ng/mL. Coagulation studies revealed no abnormalities. A urinalysis showed 14 to 16 red blood cells/high-powered field and 4g of protein. The urine culture was negative. Serology test results were negative for hepatitis C virus, hepatitis B virus, and human immunodeficiency virus. The polymerase chain reaction (PCR) assay for detecting CMV was positive with 31,400copies/mL.His chest radiograph showed a bilateral consolidation and pleural effusion (Figure 1). His pulmonary angiography, ventilation-perfusion, and abdominal ultrasound scan did not show any abnormalities. A bronchoscopy procedure was rejected due to his respiratory conditions. So that cytology or immunohistochemical tests were not possible.The patient was treated with meropenem, levofloxacin and caspofungin on his admission. Signs of acute respiratory distress and necessity of non-invasive continuous positive airway pressure and hemodialysis marked the initial clinical evolution. Follow-up chest radiographs showed progression of the findings, with irregular, multifocal, patchy consolidation (Figure 2). On the third day, treatment with vancomycin and ganciclovir was started as well. All antibacterial and antifungal drugs were administrated until his cultures were negative and a PCR assay for detecting CMV was positive. An intravenous infusion of 5mg/dl ganciclovir every 12 hours for 21 days was the specified treatment course. Viral load suppression during the first several weeks of antiviral therapy, associated with clinical and renal improvement, which allowed the stopping of hemodialysis, was finally observed. Viral load monitoring with weekly CMV-PCRs was performed for four weeks until the results were negative.

He was finally discharged after thirty days and continues to do well.

## Discussion

Idiopathic membranous nephropathy (iMN) is one of the most common causes of nephrotic syndrome in adults [1,2]. The clinical course of patients with iMN is quite variable. Spontaneous remission incidence ranges from 30 to 60% [3,4]. In contrast, the other two thirds will generally divide equally into either persistent proteinuria with longterm preservation of renal function or slow progression to renal failure [3,5].

Therapeutic interventions in patients with iMN are based on several algorithms [1,2,5]. The conservative approach should be recommended only for those patients that show a clear and progressive proteinuria decrease during the first year, together with a stable renal function. On the other hand, immunosuppressive therapy is advised without delay for high-risk patients, and recommended for medium-risk patients when nephrotic-range proteinuria persists for more than six months [2]. Our patient did not develop spontaneous remission, and immunosuppressive therapy had to be started because of nephrotic syndrome persistence and rapid worsening of renal function.

The efficacy of corticosteroids in combination with cytotoxic drugs such as chlorambucil or cyclophosphamide has been demonstrated in several studies [1,6-8]. We did not choose an anticalcineurinic drug because of renal insufficiency in our patient, and so CLB was chosen. CLB is generally well-tolerated and the most common toxicities include neutropenia and thrombocytopenia; lung toxicity is rare.

The differential diagnosis of pulmonary infiltrates in our patient may be divided into infectious and non-infectious etiologies, such as drug-induced lung disease.

The diagnosis of drug-induced pulmonary fibrosis is made after the exclusion of other etiologies for the clinical presentation, especially infection. The clinical and radiographic features are non-specific. Symptoms from drug toxicity occur from several days to 72 months after the initiation of therapy. It is known that there is no direct correlation between the dose or duration of therapy and the incidence of lung toxicity [9]. Doses of CBL ranging from 540 to 8340mg have been reported [10]. The cumulative dose of CBL of the patient we present was 220mg.

On the other hand, Torres *et al.*[11] observed side effects of chlorambucil in 47% of treated patients, most accounted for by infections (32%). Our patient was immunocompromised because of the treatment (CBL and corticosteroids) and membranous nephropathy itself. The spectrum of potential pathogens known to cause pulmonary infections in immunocompromised patients includes bacterial, fungal, and virus. Apart from clinical and radiological imaging, cultures, an antibodies test, and nuclear acid detection allow the diagnosis. Our patient had negative cultures and a PCR assay that was positive for CMV. All patients with a viral load of more than 20,000copies/mL developed CMV disease. An antibodies test for CMV has no role in diagnosing CMV disease in immunocompromised patients. This is the reason why it was not performed in this case. Clinical improvement linked to viral load suppression after a specific treatment was initiated was persuasive for the diagnosis of CMV pneumonitis.

## Conclusions

The most likely cause for pulmonary infiltrates in patients with CBL therapy appears to be treatment with CBL. However, the myelosuppressive effects of CBL enable opportunistic pneumonia viruses such as CMV, as described in our report. Therefore, we believe CMV infection should be part of the differential diagnosis of patients with respiratory symptoms treated with CBL.

## Consent

Written informed consent was obtained from the patient for publication of this case report and accompanying images. A copy of the written consent is available for review by the Editor-in-Chief of this journal.

## Abbreviations

CBL: Chlorambucil; CMV: Cytomegalovirus; iMN: Idiopathic membranous nephropathy; PCR: Polymerase Chain Reaction (PCR).

## Competing interests

The authors declare that they have no competing interests.

## Authors’ contributions

MDS and MA performed the renal biopsy. MDS, MT, MA, and AM performed the diagnosis, therapeutic management and monitoring of patient. They interpreted diagnostic tests, performed the literature search and were responsible for the therapeutic management of the patient. MT carried out the case description. All authors read and approved the final manuscript.
